# Comparison of Optic Nerve Sheath Diameter Measurements in Coronary Artery Bypass Grafting Surgery with Pulsatile and Non-Pulsatile Flow

**DOI:** 10.3390/medicina61050870

**Published:** 2025-05-09

**Authors:** Leyla Kazancıoğlu, Şule Batçık

**Affiliations:** Department of Anesthesiology and Reanimation, Faculty of Medicine, Recep Tayyip Erdoğan University, 53020 Rize, Türkiye; sule.batcik@erdogan.edu.tr

**Keywords:** pulsatile flow, optic nerve sheath diameter, ultrasonography, intracranial pressure, non-pulsatile flow

## Abstract

*Background and Objectives:* In coronary artery bypass grafting (CABG) surgeries, monitoring intracranial pressure (ICP) is crucial due to neurological risks. Although pulsatile flow (PF) during cardiopulmonary bypass (CPB) is considered more physiological than non-pulsatile flow (NPF), its impact on ICP remains unclear. This study aimed to compare preoperative and postoperative optic nerve sheath diameter (ONSD) measurements between PF and NPF techniques to evaluate their effect on ICP changes. *Materials and Methods:* Sixty patients undergoing elective CABG (aged 45–75 years, ASA II-III-IV) were enrolled and divided into two groups depending on the cardiopulmonary bypass technique determined by the surgeon: PF (Group P, n = 30) and NPF (Group NP, n = 30). ONSD measurements were performed with ultrasound before surgery (Tpreop) and after surgery (Tpostop). Hemodynamic parameters and jugular and carotid vessel diameters were also recorded. Statistical analysis included *t*-tests, Mann–Whitney U-tests, chi-square tests, and Pearson correlation. *Results:* Both groups demonstrated significant increases in ONSD postoperatively compared to preoperative values (*p* < 0.001). However, no statistically significant difference in the magnitude of ONSD change was observed between the PF and NPF groups (*p* > 0.05). Group P showed lower ejection fractions and higher total inotrope requirements compared to Group NP (*p* < 0.01), but these factors did not translate into differences in postoperative ICP dynamics. *Conclusions:* ONSD measurements increased significantly after CABG surgery, regardless of perfusion type. PF and NPF strategies were comparable in terms of their effects on ICP as reflected by ONSD changes. ONSD ultrasonography appears to be a simple, rapid, and non-invasive tool for perioperative ICP monitoring in cardiac surgery. Further studies are needed to confirm these findings with dynamic intraoperative monitoring and neurocognitive assessments.

## 1. Introduction

In coronary artery bypass grafting (CABG) surgeries, the type of flow employed by the extracorporeal support system plays a crucial role in maintaining hemodynamic stability. Although non-pulsatile flow (NPF) techniques are still commonly used, pulsatile flow (PF) more closely mimics physiological cardiac output and may offer additional systemic benefits [1]. CABG procedures can induce significant physiological alterations, and PF has been associated with improved pulmonary, hepatic, and renal functions, as well as reduced requirements for inotropic support [2]. Furthermore, PF has been shown to decrease pulmonary vascular resistance, minimize edema formation through the increased release of vasoactive substances, and enhance microcirculatory flow compared to NPF [3].

Despite these systemic advantages, neurological complications remain a major concern in CABG surgery. Factors such as venous occlusion, embolic phenomena, carotid stenosis, and technical challenges related to cannulation may predispose patients to intracranial hypertension [4]. Monitoring intracranial pressure (ICP) is critical, as elevated ICP (>20 mmHg) is associated with adverse neurological outcomes, including brain ischemia and brainstem herniation [5]. Although invasive ICP monitoring remains the gold standard, it carries significant risks such as infection, bleeding, and procedural delays. Consequently, non-invasive techniques have gained prominence. These include cranial computed tomography, magnetic resonance imaging, transcranial Doppler, intraocular pressure measurements, venous ophthalmodynamometry, and, notably, optic nerve sheath diameter (ONSD) ultrasonography [6]. Among these, ONSD sonography stands out as a non-invasive, repeatable, and easily accessible bedside method. Recent meta-analyses have demonstrated that ONSD sonography has high diagnostic accuracy for detecting elevated ICP, with pooled sensitivity and specificity values exceeding 85% [7,8]. Berhanu et al. emphasized that applying higher ONSD cut-off values (5.6–6.3 mm) enhances specificity while maintaining acceptable sensitivity [7]. Similarly, Xu et al. confirmed that ONSD ultrasonography is an effective tool for predicting intracranial hypertension, particularly in patients with traumatic brain injury, proposing an optimal cut-off value around 5.8 mm [8].

Given the risk of elevated ICP during and after CABG surgery, non-invasive monitoring methods such as ONSD measurement could play a crucial role in perioperative neurological assessment. Accordingly, this study aimed to evaluate the differences between the effects of pulsatile and non-pulsatile perfusion strategies on intracranial pressure changes during CABG surgery by evaluating preoperative and postoperative ONSD measurements.

## 2. Materials and Methods

**Figure 1:**
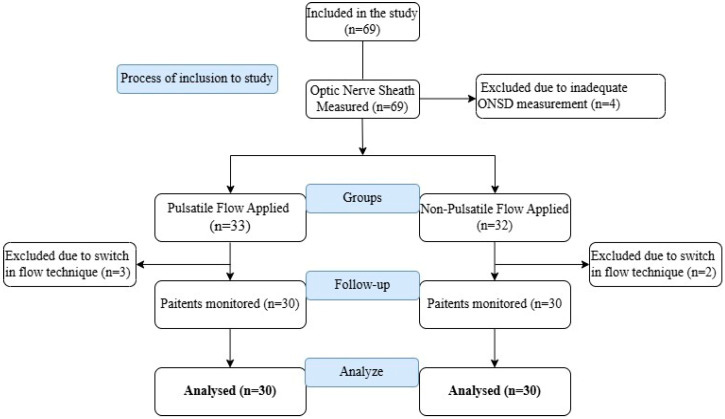
Flow chart.

The study protocol was reviewed and approved by the institutional ethics committee (2021/154). Prior to participation, all patients signed a written informed consent form. Patients aged 45–75 years who underwent elective CABG surgery and were ASA (American Society of Anesthesiologist) II–III–IV were included. Of the 69 patients initially enrolled, 5 were excluded due to intraoperative conversion from NPF to PF, and 4 were excluded because ONSD measurements could not be optimally performed (Figure 1). Data obtained from 60 participants were analyzed.

All surgical procedures were performed by three board-certified cardiac surgeons with more than 10 years of experience. Cardiopulmonary bypass procedures were managed by two certified perfusionists with over 8 years of experience. Patients were allocated into two groups based on the CPB technique: PF (Group P, n = 30) and NPF (Group NP, n = 30). The choice of flow type and surgical strategy, including graft type and number, was determined by the operating surgeon according to the patient’s clinical profile. Graft sources (left internal mammary artery, saphenous vein, or radial artery) were selected at the discretion of the surgeon based on coronary anatomy and vessel quality.

The primary outcome was to compare the effects of pulsatile (PF) and non-pulsatile (NPF) cardiopulmonary bypass techniques on preoperative and postoperative optic nerve sheath diameter (ONSD) measurements in coronary artery bypass grafting (CABG) surgeries and to evaluate changes in intracranial pressure (ICP).

The secondary outcomes were to evaluate perioperative hemodynamic parameters, including peripheral oxygen saturation, cerebral oxygen saturation, mean arterial pressure, and regional cerebral oxygenation measured by near-infrared spectroscopy (NIRS); to examine the relationship between ONSD measurements and the diameters of the jugular vein and carotid artery; and to assess postoperative changes in ICP.

Patients with known dementia, Alzheimer’s disease, cerebrovascular accident, carotid stenosis, psychiatric illnesses, emergent or redo surgeries, congestive heart failure, chronic renal failure, sepsis, hypoxia, ocular trauma, optic nerve pathology, or any neurological involvement were excluded from the study.

All ONSD measurements were performed by a single anesthesiologist with expertise in ultrasonographic neuromonitoring, who was blinded to the assigned perfusion technique. Routine intraoperative monitoring was applied. Following anesthesia induction (0.1 mg/kg midazolam, 1–2 µg/kg fentanyl, 2–3 mg/kg propofol, and 1 mg/kg rocuronium), patients were intubated and mechanically ventilated with a 50% oxygen–air mixture, tidal volume of 6 mL/kg, and ETCO2 maintained between 30 and 35 mmHg.

Potassium concentration in the cold cardioplegia solution used for myocardial protection was approximately 20 mmol/L. Cardioplegia was administered intermittently every 20 min.

Perioperative hemodynamic parameters were recorded before (Tpreop) and after surgery (Tpostop).

Demographic and hemodynamic parameters such as gender, left jugular vein and carotid artery diameters, ejection fraction, number of grafts, anesthesia and surgery duration, mechanical ventilation time, ICU stay, peripheral oxygen saturation, MAP, FiO2, NIRS values, hematocrit, lactate, cross-clamp and pump durations, fluid balance, blood product use, and right/left ONSD measurements were recorded at both time points.

### 2.1. Cardio Pulmonary Bypass Technique in Pulsatile and Non-Pulsatile Flow

Before aortic and right atrium cannulations, patients were given 300–400 IU kg^−1^ heparin to ensure that activated clotting time levels were greater than 400 s. A roller pump (Baxter Healthcare, Ann Arbor, MI, USA) and a membrane oxygenator (Dideco Compact Flo Evo, Mirandola, Italy) were used. The prime solution contained Ringer’s lactate solution (1000–1500 mL). Pump flow was adjusted to 2.2–2.4 L·m^−2^ to sustain a mean arterial pressure of 50–70 mmHg. Controlled cooling was applied to achieve a body temperature of 30 °C. Intermittent cardioplegia technique (every 20 min) was applied for myocardial protection, and a cold (4 °C), topical isotonic solution was applied to the surface of the heart. In Group P, pulse was obtained by creating temporary changes in the roles of the arm velocities.

### 2.2. Optic Nerve Sheath Diameter Measurements

Intraoperative measurements were made with ultrasound units and a linear probe in the range of 7.5–10 MHz while the patient was in the supine position. The transducer was placed on the eyelids in a sterile manner, taking care not to apply pressure, and the right and left ONSD measurements were made by adjusting the depth on the USG device to 4–5 cm. The G*Power 3.1.3 software was utilized for sample size estimation (Heinrich-Heine-Universitat Dusseldorf; Dusseldorf, Germany). In the analysis, when the effect size was determined as 0.8, the confidence interval as 95%, and the test power as 90%, it was calculated that there should be a minimum of 30 patients for each group.

### 2.3. Statistical Analysis

Data were analyzed using SPSS version 22 (IBM Corp., Armonk, NY, USA). The normality of data distribution was evaluated using the Kolmogorov–Smirnov test. Normally distributed data were reported as mean ± standard deviation and compared using the Student’s *t*-test for independent samples. Categorical data were expressed as counts and percentages and compared using the chi-square test. Although ONSD measurements were performed pre- and postoperatively, paired sample analysis (e.g., paired *t*-test or Wilcoxon signed-rank test) was not applied due to differences in data variance and normality assumptions between the two time points. Pearson correlation was used to evaluate associations between continuous variables. A *p*-value of <0.05 was considered statistically significant.

## 3. Results

The mean age of patients in Group NP was 62.6 ± 6.4 years, while the mean age in Group P was 62.4 ± 6.3 years. Preoperative data showed that ejection fraction was lower in Group P compared to Group NP (*p* = 0.008, *p* < 0.01). Intraoperative and postoperative data showed that the total inotrope dose administered was higher in Group P compared to Group NP (*p* = 0.008, *p* < 0.01) (Table 1). No statistically remarkable difference was found between the two groups in other intraoperative and postoperative data. (*p* > 0.05) (Table 1).

In Group P; right ONSD was measured as Tpreop: 4.8 ± 0.8 mm; Tpostop: 5.3 ± 0.8 mm (*p*: 0.000, r: 0.584^++)^, left ONSD was measured as Tpreop: 4.8 ± 0.7 mm; Tpostop: 5.3 ± 0.9 mm (*p*: 0.000, r: 0.736^++^). There was a positive significant relationship between Tpreop and Tpostop values in right and left ONSD measurements.

In Group NP, right ONSD Tpreop 4.9 ± 0.9 mm and Tpostop 5.2 ± 0.1 mm (*p*: 0.000, r: 0.610^++^) and left ONSD Tpreop 5.1 ± 1 mm and Tpostop 5.2 ± 1.1 mm were measured. A positive significant correlation was found in Tpreop and Tpostop values in right ONSD measurements. No statistically remarkable difference was found in Tpreop and Tpostop time periods in right and left ONSD measurements in Group P and Group NP (*p* > 0.05) (Table 2).

## 4. Discussion

The main finding of this study was that optic nerve sheath diameter (ONSD) significantly increased postoperatively in both pulsatile flow (PF) and non-pulsatile flow (NPF) groups during coronary artery bypass grafting (CABG) surgery, without a significant difference between the two groups. This suggests that both perfusion strategies are associated with postoperative changes in intracranial pressure (ICP), as reflected by ONSD measurements, regardless of flow type.

In CABG surgeries, mean arterial pressure may change according to different flow techniques. As a result of all these changes, cerebral circulation may be affected. CPB use may involve some risks. It is usually associated with organ dysfunction after surgery [9]. Strategies have been developed to protect organs, and PF in CPB is one of them. In this flow, which is thought to be more physiological, arterial pulse can be imitated. The arterial pump slows down and speeds up, creating pulsatility. There is evidence that PF reduces systemic inflammatory response and protects pulmonary, hematological, renal, cerebral, and myocardial functions, or vice versa [10,11,12,13,14,15,16]. This research focused on investigating the ICP effects of PF on ONSD. In our study, ONSD values were similar between Group P and Group NP. However, Tpostop measurement values increased in both Group P and Group NP compared to Tpreop. Therefore, this led us to the conclusion that ONSD, which is simple and easy to apply, should be considered as an additional method in the monitoring of intracranial pressure.

There are studies evaluating postoperative cognitive dysfunction related to intracranial pressure in cardiac surgery. Indeed, according to Öztürk et al., no difference was observed between the patients in the Mini Mental State Examination performed on the 3rd postoperative day after PF and NPF [14]. On the other hand, Aykut et al. administered the Montreal Cognitive Assessment test and reported a statistically remarkable improvement in the scores of the PF group 1 month postoperatively [15]. The findings of a retrospective study [16] indicated that PF was an independent predictor of cerebrovascular accidents after statistical analysis corrected for differences in demographic factors between the groups. Consequently, the increase in turbulent blood flow due to the creation of cyclic shear stress and strain by PF may be a contributing factor. Some studies reported that the type of flow did not make a difference in cerebral oxygenation recorded by NIRS regardless of whether an intraorthostatic balloon pump or roller pump was applied [17,18]. Similarly, there was no significant difference in cerebral injury markers (S-100β protein, neuron-specific enolase) [2]. In the context of our research, there was no difference between the groups in cerebral oxygenation recorded with NIRS. However, in Group P, the ejection fraction of the patients was lower and the total inotrope dose was higher. We thought that all these values could affect cerebral perfusion.

There are studies on ONSD measurements in cardiac and non-cardiac surgeries. In our literature review, we did not find ONSD measurements in PF in CABG. Ertl M et al. [19] investigated whether increased spinal cerebrospinal fluid (CSF) pressure due to ischemia-related edema of the spinal cord after thoracic endovascular aortic repair (TEVAR) caused an increase in CSF pressure defined by the expansion of ONSD and found that ONSD measurements increased compared to baseline values after the procedure. Therefore, they concluded that ONSD can be used as a marker for increased intraspinal pressure and its treatment. Similarly, Cardim D et al. [20] In hypoxic ischemic brain injury, increased ICP may cause secondary ischemic brain injury and may result in brain death. They hypothesized that invasive ICP monitoring may be risky in these patients. They reported that ONSD measurements, which are one of the non-invasive methods, can be used in the detection of brain death like invasive methods. In a pilot study including 21 patients who underwent CABG, mitral and aortic valve replacement, ICP changes in patients during the extracorporeal circulation process were monitored with ultrasound-guided ONSD measurement. For all patients, ONSD values for the right and left eyes were expressed as significantly increased compared to the initial value. They interpreted that these measurements could be useful in their follow-up [21]. We also found similar results for both groups in our study. We assume that it may be related to the variability of the demographic and hemodynamic data of the patients.

There are studies investigating the imaging of intravascular volume status with ultrasound in critically ill patients and after surgery [22,23]. In a study examining the relationship between ONSD and volume status after cardiac surgery [24], central venous pressure and 72 h net fluid balance were monitored, ONSD and inferior vena cava diameter were measured by ultrasound, and the change in ONSD was associated with the change in volume status. They concluded that it helps in improving prognosis by predicting brain edema and volume status. A similar examination was conducted in our study. However, no significant relationship was determined between the left jugular interna vena, dextra diameters, left carotid artery, dextra diameters, and ONSD measurements in both groups.

Although ONSD ultrasonography is considered a promising tool for the non-invasive detection of elevated intracranial pressure (ICP), its routine clinical integration requires careful consideration. The technique is inherently operator-dependent, and while studies suggest that a short-focused training period may be sufficient for competency, interobserver variability remains a concern without adequate experience [25]. From an equipment perspective, ONSD measurements can be performed using a standard linear ultrasound probe (7.5–10 MHz), which is widely available in most anesthesiology and intensive care settings, facilitating broader adoption [26]. However, the method does not currently support continuous automated monitoring; instead, serial measurements at predetermined intraoperative intervals are necessary to assess dynamic ICP fluctuations. Despite these limitations, the accessibility, bedside applicability, and diagnostic utility of ONSD make it a valuable adjunct in perioperative neuromonitoring, particularly in centers with basic ultrasound capabilities and trained personnel [27].

### Study Limitations

This study has several limitations that should be acknowledged. First, patients were allocated to the pulsatile and non-pulsatile flow groups based on the operating surgeon’s routine preference rather than through strict randomization, potentially introducing selection bias. Second, ONSD measurements were obtained only at preoperative and postoperative time points; intraoperative intermittent assessments were not performed, which limits understanding of dynamic cerebral perfusion changes during surgery. Third, cognitive function evaluations, such as the Mini Mental State Examination (MMSE) or Montreal Cognitive Assessment (MoCA), were not conducted, restricting the ability to correlate ONSD alterations with neurocognitive outcomes. Fourth, the study was performed at a single institution with a limited number of participants, which could restrict the applicability of the results to broader populations. Finally, long-term neurological outcomes were not assessed, highlighting the need for future studies incorporating continuous intracranial pressure (ICP) monitoring and comprehensive neurocognitive evaluations to better elucidate the clinical implications of ONSD changes after CABG surgery.

## 5. Conclusions

In this study, optic nerve sheath diameter (ONSD) measurements increased significantly after coronary artery bypass grafting (CABG) surgery, regardless of whether pulsatile or non-pulsatile flow cardiopulmonary bypass techniques were used. The type of perfusion flow did not result in a statistically significant difference in postoperative ONSD changes, suggesting that flow modality may not significantly impact intracranial pressure (ICP) dynamics during CABG. These findings support the use of ONSD ultrasonography as a simple, rapid, and non-invasive tool for indirectly monitoring intracranial pressure changes in cardiac surgery patients. Further prospective studies with intraoperative monitoring and cognitive function assessments are warranted to validate and expand upon these results.

## Figures and Tables

**Table 1:** Demographic and hemodynamic data.

	Group NP (n = 30)	Group P (n = 30)	*p* Value
**Preoperative data**			
Gender, M/F	20/10	19/11	0.791
Age, y	62.6 ± 6.4	62.4 ± 6.3	0.591
BMI, kg/m^2^	27.1± 3.0	26.3 ± 4.2	0.424
ASA Score (II/III/IV)	8/18/4	7/19/4	0.975
VJI sinistra/dextra diameter, mm	11.2 ± 1.6/10.7 ± 1.7	11.2 ± 1.5/10.5 ± 1.7	0.499/0.627
CA sinistra/dextra diameter, mm	7.3 ± 1.2/7.4 ± 1.1	7.3 ± 1.1/7.2 ± 1.2	0.700/0.942
Ejection fraction, %	50.2 ± 3.5	48.3 ± 2.7	0.008 *
**Intraoperative data**			
Number grafts, n	2.6 ± 0.4	2.5 ± 0.5	0.605
Anesthesia duration, min	149 ± 21.2	147.2 ± 18.2	0.777
Surgery duration, min	118 ± 15.8	120 ± 17.2	0.864
MV Duration, h	5.9 ± 0.7	5.8 ± 0.7	0.665
ICU stay duration, h	19.9 ± 2	20 ± 2	0.777
SpO_2_, %	97.7 ± 1.8	97.7 ± 1.9	0.502
MAP, mmHg	68.8 ± 6.1	66.6 ± 9.5	0.067
rScO_2_ right/left, %	65.5 ± 6.4/65.9 ± 7.7	63.8 ± 6.1/631 ± 7.7	0.573/0.378
Total inotrop dosage, µg/min	3.2 ± 4.1	6.2 ± 5.1	0.008 *
Hematocrit	29.5 ± 4.4	28.4 ± 4.4	0.728
Lactate, mmol/L	1.7 ± 0.4	1.7 ± 0.5	0.818
Cross clamp duration, min	56.8 ± 15.5	57.0 ± 14.6	0.965
Pump duration, min	94.8 ± 21.0	96.6 ± 16.2	0.534
Pump liquid balance, ml	813.6 ± 421	1000 ± 577	0.206
Blood products given, ml	492.0 ± 276	685.3 ± 407	0.079

Group NP: non-pulsatile flow group, Group P: pulsatile flow group, BMI: body mass index, VJI: veno jugilaris interna, CA: carotis arter, MV: mechanical ventilation, ICU: intensive care unit, SpO_2_: peripheral oxygen saturation, MAP: mean arterial pressure, rScO_2_: regional cerebral oxygen saturation. Values are expressed as mean ± standard deviation. * *p* < 0.05.

**Table 2:** Optic nerve sheath diameter measurements.

Group	Right Eye ONSD, mm				Left Eye ONSD, mm			
	Tpreop	Tpostop	*p*	r	Tpreop	Tpostop	*p*	r
Group NP	4.9 ± 0.9	5.2 ± 0.1	<0.001	0.610 ^++^	5.1 ± 1.0	5.2 ± 1.1	<0.001	0.386
Group P	4.8 ± 0.8	5.3 ± 0.8	<0.001	0.584 ^++^	4.8 ± 0.7	5.3 ± 0.9	<0.001	0.736 ^++^

ONSD: optic nerve sheath diameter, Group NP: non-pulsatile flow group, Group P: pulsatile flow group, Tpreop: preoperative, Tpostop: postoperative, r ^++^: positive significant relationship.

## Data Availability

Dataset available from the authors upon request.

## Abbreviations

The following abbreviations are used in this manuscript:

ASA	American Society of Anesthesiologist
BMI	Body Mass Index
CA	Carotis Arter
CABG	Coronary Artery Bypass Grafting
CPB	Cardiopulmonary Bypass
CSF	Cerebro Spinal Fluid
CT	Cranial Tomography
IBM	International Business Machines
ICP	Intracranial Pressure
ICU	Intensive Care Unit
MAP	Mean Arterial Pressure
MV	Mechanical Ventilation
NIRS	Near-Infrared Spectroscopy
NPF	Non-Pulsatile Flow
ONSD	Optic Nerve Sheath Diameter
PF	Pulsatile Flow
SD	Standard Deviation
SPSS	Statistical Package for the Social Sciences
TEVAR	Thoracic Endovascular Aortic Repair
USG	Ultrasonography
VJI	Veno Jugilaris Interna
